# Moisture Content Detection in Mango (*Mangifera indica* L., cv. Ataulfo) and Papaya (*Carica papaya*) Slices During Drying Using an MMI-Based Sensor

**DOI:** 10.3390/s25226902

**Published:** 2025-11-12

**Authors:** Guadalupe López-Morales, Yuliana M. Espinosa-Sánchez, Ariel Flores-Rosas, Héber Vilchis

**Affiliations:** 1 Instituto de Investigación e Innovación en Energías Renovables, Universidad de Ciencias y Artes de Chiapas, Tuxtla Gutiérrez 29039, Chiapas, Mexico; guadalupe.lopez@unicach.mx (G.L.-M.); heber.vilchis@unicach.mx (H.V.); 2 Facultad de Ciencias en Física y Matemáticas, Universidad Autónoma de Chiapas, Tuxtla Gutiérrez 29055, Chiapas, Mexico; ariel.flores@unach.mx

**Keywords:** multimode interference, optical fiber sensor, moisture content detection, non-destructive sensing

## Abstract

Monitoring moisture content in agricultural products during the drying process is critical for ensuring quality, preserving nutritional value, and optimizing energy consumption. This study presents the design and implementation of an optical fiber sensor based on multimode interference (MMI) for non-destructive detection of moisture content in mango (*Mangifera indica* L., cv. Ataulfo) and papaya (*Carica papaya*) slices during convective drying at 57 °C. Two sensors were designed and fabricated: one operates in the 975 nm range and the other in the 1414.25 nm range. These sensors detect variations in the refractive index caused by moisture loss, which directly affects the MMI spectral response. The sensor output was correlated with reference gravimetric measurements, demonstrating a dependence in tracking the output power as a function of the reduction in humidity over time. The results confirm the feasibility of the MMI-based optical fiber sensor as a reliable tool for in situ monitoring of drying dynamics in tropical fruits, offering potential applications in agri-food processing and quality control.

## 1. Introduction

In recent decades, the development of food drying and dehydration techniques has increased due to the growing demand for healthy, easy-to-transport foods with longer shelf lives that also retain their nutritional and sensory properties [1]. These processes are fundamentally thermophysical and involve the transfer of heat to the product to facilitate moisture removal through the diffusion of water vapor. The primary objective of drying agricultural commodities is to reduce their moisture content to safe levels that inhibit microbial growth, thereby extending shelf life while preserving quality [2].

Drying and dehydration processes are widely regarded as natural, sustainable, and environmentally friendly preservation alternatives, particularly when using solar dryers, that offer advantages to both producers and consumers. Their simplicity, low cost, and energy efficiency make them particularly suitable for rural areas and small-scale agricultural operations. In regions with a rich diversity of fruit crops, such as Chiapas, México, where production is focused on highly perishable fruits, including mango (*Mangifera indica* L., cv. Ataulfo), papaya (*Carica papaya*), plátano (*Musa paradisiaca*) and pitahaya (*Hylocereus undatus*), the application of these techniques plays a vital role. By extending shelf life, retaining nutritional and functional properties, and minimizing postharvest losses, these methods not only reduce food waste but also enhance the commercial value of the final product [3,4,5].

To ensure that dried and dehydrated fruits are of high quality and have a long shelf life, it is essential to implement specific process controls that reduce the variability in the final products. These controls help meet both legislative standards and consumer expectations [6]. Therefore, in conjunction with drying techniques, it is necessary to implement sensors that help monitor the amount of water present during this process. The concept of water content (WC) refers to the total amount of water present in a sample, while moisture content (MC) refers to the amount of water relative to the amount of dry matter.

Several methods can measure moisture content during a fruit drying process, such as gravimetric methods and rapid/instrumental techniques, to name just a few [7]. Each has its own advantages, limitations, and suitability depending on the product type and the process conditions, while the most common method is gravimetric analysis, where sample weight is measured before and after drying, is used as reference techniques due to their high accuracy, they are slow, destructive, and unsuitable for real-time process control. Similarly, chemical approaches like Karl Fischer titration and thermogravimetric analysis (TGA), although highly precise, are labor-intensive and impractical for continuous monitoring [8].

To address the need for real-time, non-destructive, and cost-effective moisture monitoring, alternative sensing technologies have been developed, including near-infrared (NIR) and terahertz spectroscopy, microwave techniques, and electrical impedance-based sensors [9,10,11]. However, these approaches often require complex calibration models, are sensitive to sample heterogeneity, or involve high equipment costs.

In particular, absorption spectroscopy in the NIR region provides valuable information on the presence of water molecules and other organic compounds. A weak absorption band is observed between 960 and 980 nm, corresponding to the first overtone of O–H stretching vibrations in water. Another weak band appears around 1190–1210 nm, attributed to the second overtone of O–H stretching. A stronger absorption peak occurs in the 1400–1600 nm range, primarily associated with O–H bonds in water. However, interactions between water and sugar molecules, particularly C–H and O–H groups, can cause peak shifts and spectral modifications in this region [9,12].

For this purpose, and as a complement to the existing methods, optical technique-based sensors can be incorporated into monitoring systems, thanks to their easy implementation, interpretation, and non-invasiveness. The use of optical fibers in the field of sensing has grown in recent years, as they play a fundamental role in the detection and measurement of various parameters. Their range of applications also includes biomedicine, environmental sensing, and mechanical and industrial measurements, among others [13]. All of this is possible because of their structure and the materials from which they are made, as they allow them to be used in extreme environmental conditions, thereby expanding their application range.

Optical fiber sensors exhibit several key advantages, such as compact dimensions, rapid response times, remote sensing capabilities, and inherent resistance to corrosive environments. Furthermore, their ability to function reliably under extreme environmental conditions establishes a robust platform for the accurate measurement of various physical, chemical, or biological parameters in a wide range of applications [14].

There are many types of optical fiber sensors, some of which have more refined optoelectronics and sophisticated designs. However, these features also make their fabrication more complex and increase the cost of the sensor. For this reason, in this work, a relatively simple sensor has been chosen—one that is easy to build and operate, and whose structure may allow future in situ measurements. This sensor is based on the multimode interference (MMI) effect, where changes in its spectrum are observed when it comes into contact with the sample.

In recent years, there has been a growing interest in the application of MMI effect within integrated optics, due to the outstanding performance characteristics of MMI-based devices. Beyond the aforementioned advantages, these devices offer ease of integration into various experimental configurations, including as ring-shaped fiber lasers, modulators, and other photonic systems [15]. MMI is used in optical fibers for applications that include beam shapers, filters, and sensors. Among these applications, the use of devices based on MMI filters has increased, as they are not only employed as filters but also used within laser cavities as saturable absorbers and as sensors [16,17,18].

In addition to their unique spectral characteristics and the high sensitivity of MMI-based sensors, their fabrication is easy, fast, and low-cost. It is worth mentioning that they have received special attention over the past decade, as they are capable of sensing both conventional physical variables such as refractive index, temperature, displacement, and strain, as well as non-conventional ones such as vibration, pressure, voltage, electric current, magnetic field, relative humidity, gas concentration, and wavelength [19,20,21,22,23].

MMI is a property of multimode waveguides. It should be clarified that the self-imaging phenomenon is a particular case of MMI, which occurs under strict high-symmetry conditions. In general, self-imaging can be described as the reproduction of the input field during propagation as a result of multimode interference. That is, if multiple modes are excited within the multimode fiber (MMF), MMI will occur. However, the presence of MMI does not necessarily imply that self-imaging will take place, as certain specific conditions must be met for it to occur. It is very common to operate MMI devices under self-imaging conditions because this ensures a spectral response similar to that of a filter when the excitation spectrum is continuous.

In any case involving MMI, the length of the multimode fiber (MMF) plays a key role in the outcome. In the case of restricted symmetric interference, individual images of the input field are produced at effective distances, described by Equation (1),

(1) $$L=p\frac{n_{EFF}D_{EFF}^{2}}{\lambda_{0}}$$

where $\lambda_{0}$ is the working wavelength, *p* is the number of self-images that can take values of 1, 2, 3, etc., $D_{EFF}$ is the effective diameter of the no-core fiber (NCF) and $n_{EFF}$ is the effective refractive index [24].

The fourth self-image (p=4) replicates the input field configuration, while the first and third images produce a broader version of it [15,25].

**Figure 1:**
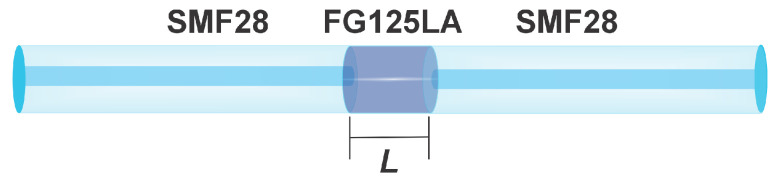
MMI sensor structure.

Conventionally, such a device consists of a multimode fiber (MMF) spliced between two single-mode fibers (SMF). However, to increase sensitivity, a no-core fiber has been used in place of a conventional MMF, as shown in Figure 1 [19]. The increased sensitivity observed when using a NCF is attributed to the enhanced interaction with the surrounding medium through the evanescent field. In other words, it is not only the multimodal self-imaging phenomenon that contributes, but also the extended evanescent field, which allows interaction with the fiber’s environment. The surrounding medium effectively acts as a cladding, and due to the large refractive index contrast between the NCF and the surrounding air, the NCF behaves equivalently to a MMF [26,27,28].

This configuration has been used for various purposes, such as measuring the refractive index of liquids and the composition of liquid mixtures, among others [29]. The guided modes supported by the NCF are excited at the SMF–NCF interface, where the input SMF launches the light, and the output SMF collects it. As previously mentioned, due to the self-imaging phenomenon, the excited modes replicate the input field at special periodic positions within the NCF [18].

In this work, we present an MMI-based sensor operating in the infrared spectral range of 975–1420 nm for the detection of water content in mango and papaya slices. The sensing mechanism relies on refractive index variations induced by moisture loss, which directly modulate the MMI spectral response. This proposed approach represents an initial step toward the development of complementary techniques for monitoring water reduction in fruits during the drying process.

## 2. Materials and Methods

### 2.1. Sample Preparation

#### 2.1.1. Water and Sugar Solution

Given that fruits are primarily composed of water, fructose, and other constituents such as vitamins, preliminary tests were conducted using liquid solutions prepared by dissolving 0.5 g of a fructose substitute (commercial stevia) in 15 mL of distilled water. The objective was to evaluate the sensor’s spectral response to pure water and, subsequently, to water–sugar mixtures at various input wavelengths, in order to assess its sensitivity to changes in the sample composition.

#### 2.1.2. Mango and Papaya Samples

A piece of fresh mango (*Mangifera indica* L., cv. Ataulfo) and papaya (*Carica papaya*) were purchased from a local supermarket in the state of Chiapas, Mexico, in August. The fruits were selected for uniform size, absence of visible defects or mechanical damage, and qualitative ripeness based on peel color. These fruits were chosen because they are perishable due to their high initial water content and because they are seasonal.

Fruits were washed with distilled water to remove surface impurities and allowed to air-dry at room temperature. Each fruit was manually peeled and sliced using a kitchen slicer. From each fruit, five slices were obtained with dimensions of approximately 2 mm thickness, 2.5 cm width, and 4 cm length, measured using a digital caliper to ensure dimensional uniformity and reproducibility across all samples.

The slicing process was carefully standardized to promote homogeneous moisture diffusion and uniform drying behavior during subsequent thermal treatment. Immediately after slicing, the fresh weight of each sample was measured using an analytical balance (Pioneer PA224, OHAUS, Parsippany, NJ, USA) with a precision of 0.01 g, and the samples were stored in clean, labeled containers prior to drying experiments. The initial average weights were as follows: mango slices, 3.0441 ± 0.0187 g and papaya slices, 3.0083 ± 0.0326 g.

### 2.2. Drying Process

Since the recommended drying temperatures in the literature indicate that they should not exceed 60 °C to avoid the degradation of volatile compounds and vitamins, the drying was conducted below this limit using a natural convection drying chamber (BLINDER) at a constant temperature of 57 °C [30,31]. The initial weight of each sample was measured, followed by measurements every 30 min to monitor mass loss. Drying was considered complete when the sample weight stabilized, which occurred around 480 min. The water content (WC), expressed as a percentage of the total initial weight, was computed using $WC=(w_{t}/w_{w})*100$%, where $w_{t}$ is the current mass weight (g) at time *t* and $w_{w}$ is the initial mass (g) of the sample. Moisture content (MC) was calculated on a dry basis using the expression $MC=\left(w_{t}-w_{d}\right)/w_{d}$, where $w_{d}$ is the final dry mass. These calculations follow the methodology described in [9].

To normalize the drying behavior across fruit types, the moisture ratio (MR) was used. MR is defined as the ratio between the moisture content at a specific time ($MC_{t}$) and the initial moisture content ($MC_{o}$) represented by the equation $MR=\left(MC_{t}-MC_{e}\right)/\left(MC_{o}-MC_{e}\right)$, where $MC_{e}$ is the equilibrium moisture content, which was assumed to be approximately zero due to negligible moisture exchange at the final stages of drying. Thus, the equation simplifies to $MR=MC_{t}/MC_{o}$.

Experimental MR data were fitted to four thin-layer drying models commonly used in food dehydration studies: Lewis, Page, Henderson and Pabis (HP), and Logarithmic (Log) models [9,30]. This is because these models offer an optimal balance between simplicity, empirical flexibility, and statistical accuracy when fitting time-dependent moisture data for a wide range of agricultural and food products, including tropical fruits such as mango and papaya, and have consistently demonstrated reliable fitting performance and high predictive power [32,33]. Model fitting was performed using nonlinear regression analysis in MATLAB R2020a (MathWorks Inc.), and the quality of fit was evaluated based on the root mean square error (RMSE) and the coefficient of determination (R^2^) for each model.

Finally, five slices at different moisture ratios were selected for further analysis using the MMI-based fiber optic sensor.

### 2.3. Measurement of the NIR Absorption Spectrum of the Samples

The spectral response in the NIR range of papaya slices with different moisture levels was measured using a UV-Vis-NIR spectrophotometer (SHIMADZU, Kioto, Japan, model UV-3600) in absorption mode, covering the range from 850 to 1650 nm with a resolution of 1 nm.

### 2.4. Sensor Preparation

The sensor structure is shown in Figure 1. It consists of an FG125LA no-core fiber spliced between two sections of single-mode fiber (SMF28, Corning Incorporated, Corning, NY, USA), which serve to couple light into the NCF and to collect the light exiting it. The length of the NCF was calculated using Equation (1).

To determine the value of *L* for each designed sensor, the operating wavelengths were selected according to the spectral response information obtained in the NIR range of the samples, where strong NIR absorbance is observed around 970 nm and 1450 nm (this information is shown in Section 3.2). Therefore, the selected wavelengths for each sensor are 1450 nm and 980 nm. Since both sensors were fabricated using the same type of NCF, the $n_{EFF}$ values are 1.444 (for 1450 nm) and 1.451 (for 980 nm), and $D_{EFF}=125$ μm in both cases, as provided by the manufacturer in the FG125LA datasheet. These values were selected because when the medium surrounding the NCF is air, the guided modes are mainly confined within the silica region due to the large refractive index contrast between air and silica. Consequently, the effective refractive index approaches that of silica, and the effective modal area becomes similar to the intrinsic cladding diameter [24,34]. Additionally, the selected value of p=1 results in L=1.556 cm for $\lambda_{0}=1450$ nm and L=2.32 cm for $\lambda_{0}=980$ nm.

Using p=4 produces a more precise self-image of the input field, resulting in a sharper spectral response. However, in this work, p=1 was chosen because, although p=1 or p=3 generate wider optical field images (and therefore broader spectra), using p=1 reduces the NCF length. This is advantageous for the present design, as L=1.556 cm or L=2.32 cm ensures direct contact between the sample and the NCF. This choice allows the development of a more compact sensor that can be easily integrated into portable or space-limited systems. Moreover, p=1 allows the formation of the first self-image and provides sufficient sensitivity to detect environmental variations, such as refractive index changes associated with the moisture content in fruits, which is the main objective of this study. Previous studies have also shown that sensors based on SMF–NCF–SMF configurations with p=1 can maintain good spectral sensitivity, particularly when the design is optimized using parameters such as fiber diameter, operating wavelength, and the type of interaction with the sample [9,29].

After completing the calculations to fabricate both sensors, the coating of the fibers was removed, the fibers were cleaned, properly cleaved, and carefully centered in the fusion splicer before performing the splicing. The splices between the SMF-28 and FG125LA optical fibers were carried out using a core-alignment fusion splicer. Both fibers have a cladding diameter of 125 µm, which ensures accurate geometric matching during the splicing process and helps minimize misalignment losses. The insertion losses indicated by the splicer were monitored, and splices with losses below 1 dB were considered acceptable due to the abrupt change in the optical structures. The losses remained consistently low (between 0.1 and 0.3 dB), owing to the matching cladding diameters, which reduce lateral and angular misalignment at the junction. All measurements were performed in a closed, temperature-controlled environment at 22 °C using air conditioning. Although humidity was not directly recorded, the climate control system maintained typical laboratory conditions (approximately 40–60% relative humidity), ensuring stability during measurements.

#### Measurement Procedure

**Figure 2:**
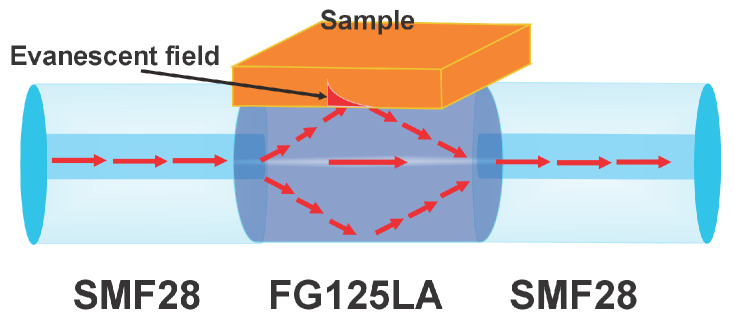
Structural diagram of the sensor with the sample, where the red arrows, shown as a ray diagram, indicate the interaction between the energy supplied by the source and the evanescent field with the sample.

In the physical operating mechanism of the sensor, one of the key aspects is the MMI that occurs within the NCF. The light injected from the SMF is guided into the NCF section, where it propagates along its length. At the first SMF/NCF interface, since the NCF has no core to confine the fundamental mode, the optical field expands within the NCF and excites several modes that propagate with different propagation constants. Along the NCF, these modes interfere constructively and destructively, producing an intensity pattern that depends on the wavelength. It is important to note that the NCF has no coating, so a portion of the evanescent field extends outside the fiber and slightly penetrates the sample that is in contact with it (Figure 2). The samples exhibit a refractive index that depends on their water content and other constituents. As the samples lose water, their refractive index increases due to the reduction of water content and the higher relative concentration of solutes [35,36]. These variations in the sample’s refractive index modify the effective refractive index of the propagating modes within the NCF, thereby altering the interference condition and consequently causing a shift in the transmission spectrum of the sensor. Finally, at the second NCF/SMF interface, the total optical field coupled into the output single-mode fiber is determined by the relative phase differences among the guided modes in the NCF, giving rise to the observed spectral response [25,27,37,38].

**Figure 3:**
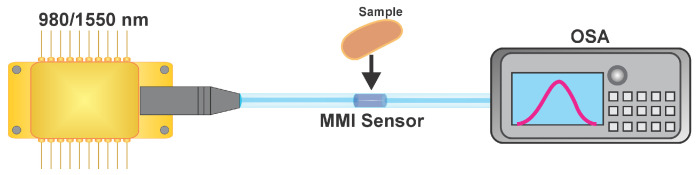
MMI sensor setup.

To experimentally verify this behavior, the following setup was implemented. First, a 980 nm laser diode (Thorlabs PL980P330J) was used as the illumination source. The sensor output was connected to an optical spectrum analyzer (OSA, Anritsu MS9740A, Anritsu Corporation, Atsugi, Japan) to visualize the spectrum during each measurement (Figure 3). It should be noted that for the sensor designed to operate at a wavelength of 980 nm, the NCF length is 2.32 cm. Subsequently, a superluminescent laser diode (Thorlabs SLD1550S-A1, Thorlabs, Inc., Newton, NJ, USA) was used to evaluate the sensor’s performance in a spectral range from 1400 nm to 1650 nm. For this range, also with p=1, the calculated NCF length was 1.536 cm.

The measurement procedure was as follows: Once the experimental setup was assembled, the spectrum of the MMI sensor was recorded before and after placing the sample on the sensor (Figure 3). For each sample, spectra were collected using a pump power of 167.5 mW for the 980 nm laser and 599.4 μW for the superluminescent laser. The samples consisted of mixtures of water with sugar, as well as slices of papaya and mango with varying moisture levels. After each measurement, the sensor was cleaned with distilled water and acetone. The reference (baseline) spectrum used for the comparison was always the one obtained with the sensor in the absence of a sample. Each measurement was repeated three times to verify the repeatability and accuracy of the experiment.

## 3. Results

This section presents the performance evaluation of the MMI-based sensor for monitoring the moisture content of mango and papaya slices during the drying process.

Section 3.1 details the weight loss curves as a function of drying time, along with the corresponding moisture content and moisture ratio values. The moisture ratio curve was fitted using four mathematical models to predict drying behavior. In addition, near-infrared absorption spectra of papaya samples at different moisture levels are presented. Section 3.2 presents the spectral output of the MMI sensor using excitation sources at 980 nm and 1550 nm for mango and papaya slices with varying moisture content. The recorded spectra illustrate the sensor response when interacting with samples at specific moisture levels.

### 3.1. Drying Curve

**Figure 4:**
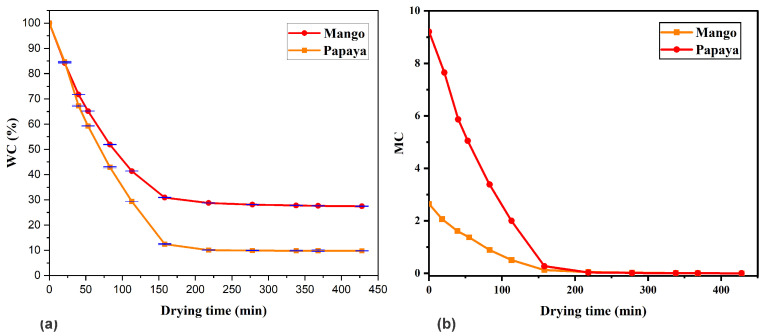
(**a**) Percentage water content and (**b**) moisture content as a function of drying time for mango (yellow line) and papaya (red line).

Figure 4a shows the percentage of water content relative to the total water content of the mango and papaya slices throughout the drying process. Since three measurements were performed for each sample, the maximum standard error obtained was 0.0124 and 0.0299 for mango and papaya, respectively, confirming the precision of the measurements. The low standard error values indicate a high degree of reliability in the recorded data, supporting the robustness of the drying kinetics and moisture content results presented in this study. Both fruits showed a continuous decrease in water content with drying time. In the initial stage of drying, before 150 min, water loss was relatively high, indicating rapid evaporation of surface moisture. As drying progressed, the rate of weight loss gradually decreased and the curve began to flatten, suggesting a reduction in moisture loss from the inner layers of the fruit to the surface. The total drying time required to reach a constant weight was approximately 320 min for papaya and 350 min for mango under the same drying conditions. The initial moisture contents were 90.21% (wet basis) for papaya and 72.55% for mango, which decreased to approximately 9.79% and 27.45%, respectively, by the end of the drying process. This indicates that the papaya had a higher water content, which could be related to the ripening time of each sample. The drying behavior followed a typical trend observed in most fruit dehydration processes, characterized by a short constant rate period followed by a prolonged falling rate period. These results are consistent with the expected drying kinetics of high-moisture tropical fruits.

The variation in moisture content (MC) of mango and papaya slices as a function of drying time is shown in Figure 4b. This parameter indicates the proportion of water in dry matter. It can be seen that papaya contains almost nine times more water than dry matter, and in the case of mango, almost three times more.

**Figure 5:**
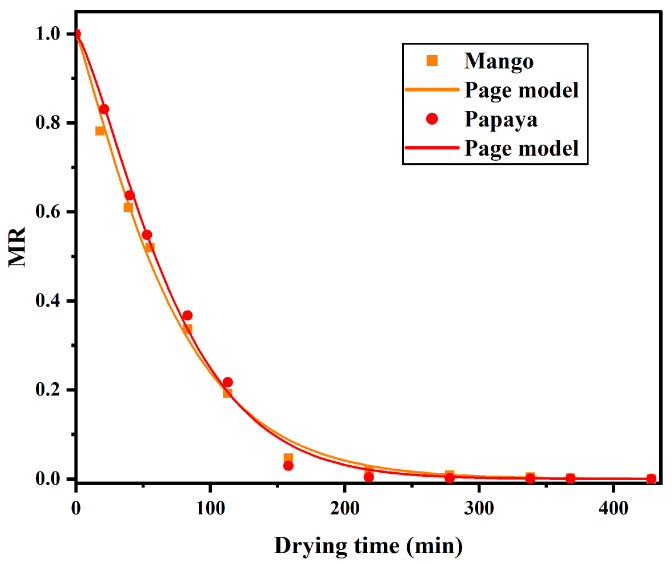
Moisture ratio for mango (orange line) and papaya (red line).

The moisture content data were later used to compute the moisture ratio (MR) and fit drying models to describe the kinetics of the process. The MR values were plotted as a function of drying time, as shown in Figure 5. The curves showed a typical exponential decay pattern, commonly observed in the drying behavior of biological materials. This behavior suggests that internal resistance to moisture transport increased over time, possibly due to the collapse or shrinkage of cellular structures. The trends of both MR curves are very similar.

To describe the drying behavior, four commonly used thin-layer drying models were applied to the experimental data: the Lewis, Page, Henderson–Pabis, and Logarithmic models. The mathematical expressions for each model are presented in Ref. [9]. Model parameters were estimated using nonlinear regression analysis, and the goodness of fit was assessed based on the coefficient of determination (R^2^) and the root mean square error (RMSE).

Table 1 summarizes the coefficients and statistical analysis of the tested models. The Lewis model, represented by a pure exponential equation, typically underestimates the moisture decay rate during the drying process, resulting in higher RMSE and lower R^2^ values, particularly for papaya slices. The Henderson and Pabis (HP) model incorporates the parameter a, which can be associated with the structural or diffusive characteristics of the material, thereby improving the R^2^ value and reducing the estimation error. The logarithmic model introduces an additional constant c that enhances the fitting accuracy by adapting to both the early and final stages of drying. However, the Page model introduces the empirical exponent n, which adjusts the curvature of the drying curve more effectively. Consequently, this model provided the best fit for both mango and papaya slices, yielding the highest R^2^ values (0.996) and the lowest RMSE values. This confirms the model’s robustness and predictive accuracy. These outcomes are in agreement with previous studies reporting that the Page model consistently provides the best fit for thin-layer drying of mango and papaya [32,33].

### 3.2. NIR Spectra Response of Papaya Sample

**Figure 6:**
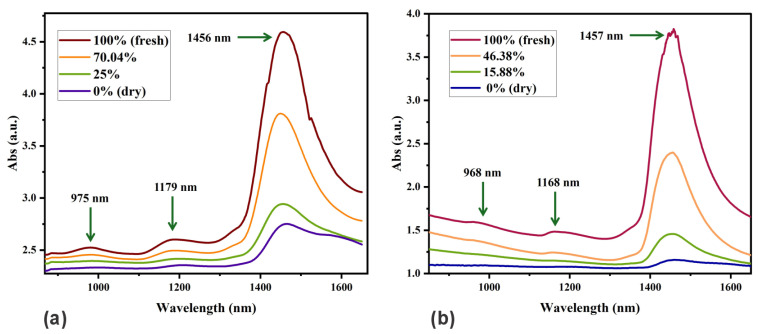
Absorbance spectra response in the NIR range with different moisture ratio level. MR values at 0% and 100% correspond to a dry and fresh sample, respectively. (**a**) Mango. (**b**) Papaya.

NIR spectra of mango and papaya slices at varying moisture content levels are displayed in Figure 6a and Figure 6b, respectively. The spectrum corresponding to the fresh sample (MR = 100%) shows three distinct absorbance peaks. A weak peak observed in the 950–1021 nm range is attributed to the second overtone of the O–H stretching vibrations in water. Another weak peak at 1110–1255 nm is likely associated with C–H and O–H overtone bands arising from sugar–water interactions. The prominent peak around 1385–1580 nm corresponds to the first overtone of O–H stretching in water, intensified by hydrogen bonding and interactions with sugar molecules, particularly those involving O–H and C–H groups. Such interactions often induce peak shifts and spectral broadening in this region. As the sample dries, a decrease in the absorbance is observed. The first two absorption peaks disappear for 0% MR. The minor variations observed in the absorption peaks among the analyzed fruits are most likely due to differences in their intrinsic physical and chemical properties, such as moisture content, sugar composition, and matrix structure. Nevertheless, the observed spectral shifts were minimal, not exceeding 10 nm. These findings are consistent with previous reports describing comparable near-infrared absorption spectra for mango and papaya [39,40].

These absorption bands are consistent with literature reports for high-water-content food matrices. Water exhibits strong NIR absorbance near 970 nm and 1450 nm due to O–H vibrations, with band broadening caused by hydrogen bonding [41,42].

These findings supported the selection of two laser sources operating in the 950–1021 nm and 1385–1580 nm ranges, as these regions exhibited significant spectral variations associated with changes in the moisture content.

### 3.3. MMI Sensor Response to Sample

Thanks to the information provided by the absorbance spectrum in the NIR range (Figure 5), where it can be observed that water shows strong absorbance near 970 nm and 1450 nm, laser sources with wavelengths close to these values were used (one at 980 nm and another with a spectral range from 1400 nm to 1650 nm, also called superluminescent) to perform the measurements. It is worth mentioning that the spectra of the MMI sensors were centered at 975.312 nm and 1414.25 nm for each laser source, respectively.

Based on the absorbance spectrum in the near-infrared (NIR) region (Figure 5), where water exhibits strong absorption bands near 970 nm and 1450 nm, two laser sources were selected for the measurements: a laser at 980 nm and a superluminescent source covering the 1520–1580 nm range. The corresponding MMI sensor spectra were centered at 975.312 nm and 1414.25 nm, respectively, for each excitation source.

#### 3.3.1. Water and Glucose Mixture

**Figure 7:**
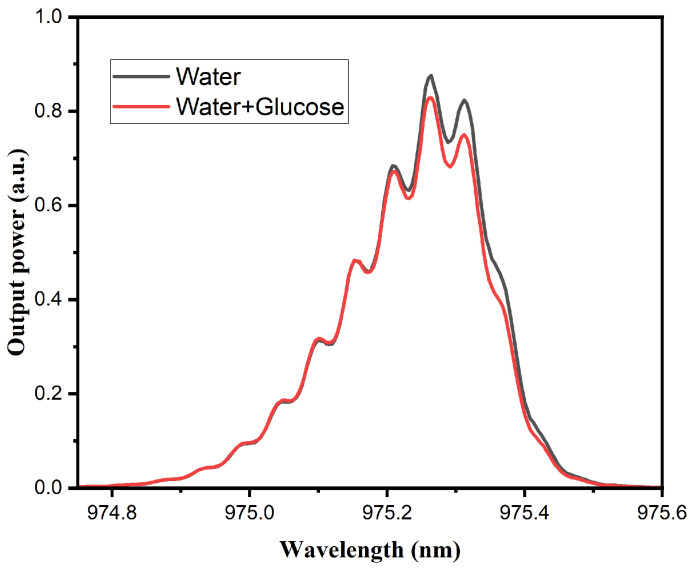
Spectral response of water (black line), and the water–glucose mixture (red line), obtained with the MMI sensor with the 980 nm laser source.

**Figure 8:**
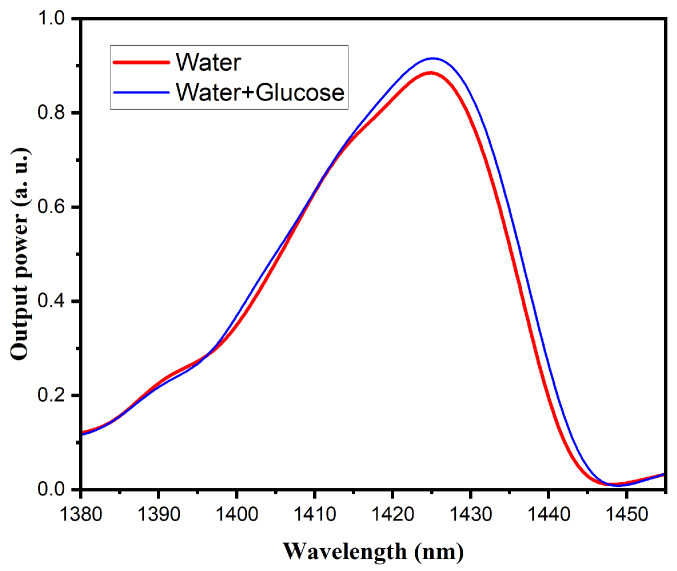
Spectral response of water (red line), and the water–glucose mixture (blue line), obtained with the MMI sensor with the superluminescent laser source.

In order to evaluate the response of the MMI sensors to variations in water and glucose content in fruits, measurements were initially performed using pure water, followed by a water–glucose solution, using the two pump sources mentioned above. Figure 7 shows the response of the MMI sensor with a 980 nm laser source, where the presence of water and sugar induces only a change in output power, without any observable spectral shift. This behavior is consistent with NIR absorption characteristics, suggesting that the response is predominantly governed by water interactions. It should be noted that the modulation observed in this and other graphs using the 980 nm laser source is not associated with water detection in the samples; rather, it is related to the longitudinal modes of the laser cavity and the interaction with the Bragg grating integrated into the laser. In contrast, the response of the sensor with the superluminescent laser source (Figure 8) exhibits both variations in output power and a noticeable spectral shift, indicating sensitivity to changes in both water and glucose concentrations.

#### 3.3.2. MMI Sensor for the 980 nm Laser Diode

**Figure 9:**
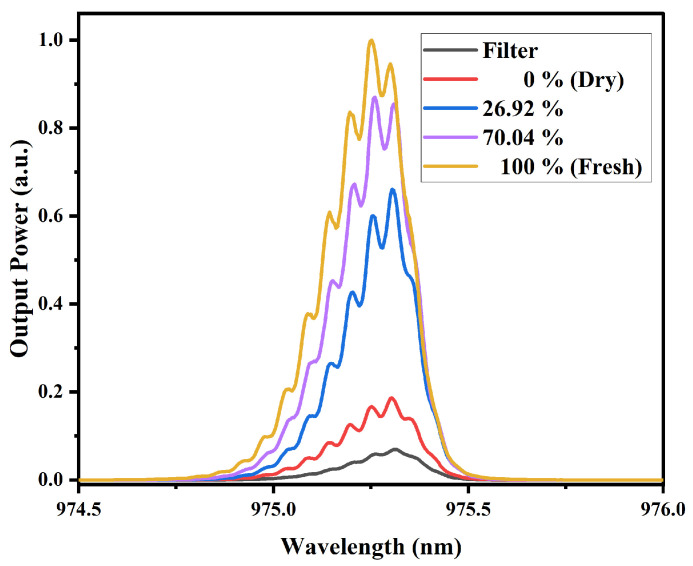
Spectral response of mango samples with different MR values, obtained with the MMI sensor with the 980 nm laser source. The dry and fresh samples correspond to 0% and 100%, respectively.

Figure 9 illustrates the spectral response of the MMI sensor under excitation with a 980 nm laser source, using mango slices with varying water content as test samples. For each sample, three measurements were performed, allowing the calculation of the standard deviation. For samples with MR values of 100%, 70.04%, 26.92%, and 0%, the standard deviation ranged from 0.0098 to 0.0172, 0.017–0.0334, 0.024–0.035, and 0.024–0.0315, respectively, showing very low dispersion and high measurement reliability. The results indicate that the output power increases proportionally with the fruit’s water content; specifically, the highest power level corresponds to fully hydrated (100%) mango slices, as indicated in the figure legend, while slight spectral broadening is observed, no significant wavelength shift is detected, suggesting that the sensor response at this wavelength is primarily influenced by absorption characteristics associated with water content.

**Figure 10:**
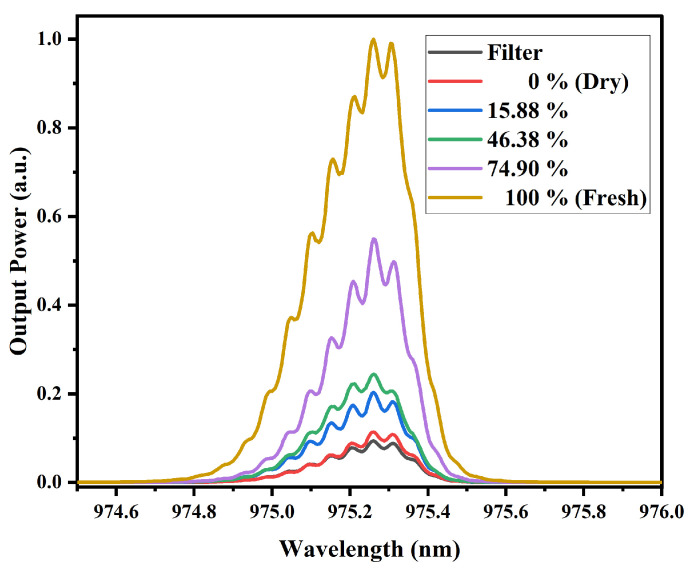
Spectral response of papaya samples with different MR values, obtained with the MMI sensor with the 980 nm laser source. The dry and fresh samples correspond to 0% and 100%, respectively.

In Figure 10, presents the spectral response of the MMI sensor for papaya slices with different water contents. The standard deviation for MR values of 100%, 74.90%, 46.38%, 15.88%, and 0% was 0.0148–0.0204, 0.0168–0.0228, 0.014–0.0325, 0.0202–0.0324, and 0.0195–0.0284, respectively, confirming the repeatability and reliability of the sensor. A behavior similar to that seen in Figure 9 is observed, that is, higher water concentrations in the samples result in a higher output power. Although some degree of spectral broadening is observed, there is no appreciable wavelength shift, indicating that the sensor’s response remains centered despite variations in moisture content.

Considering the structural characteristics of mango slices, which have a higher fiber content and a lower initial moisture content compared to papaya, it can be observed that the sensor output power is higher for mango. This indicates a lower optical power absorption. Such behavior is consistent with the near-infrared absorption mechanism, where higher water content results in stronger absorption of NIR radiation.

It is important to highlight that, for both types of samples exhibit similar behavior, particularly characterized by the absence of spectral shifts and the changes occur only in the output power. This indicates that the variations detected by the sensor (around this wavelength and according to the NIR spectra obtained) are due to attenuation changes or optical losses caused by the samples. In other words, the MMI sensor, when pumped at this wavelength, demonstrates low sensitivity to refractive index variations and instead detects water content through changes in transmitted power. Furthermore, a clear relationship between absorbance and the sensor’s output power is also observed.

#### 3.3.3. MMI Sensor for the Superluminescent Laser Diode

**Figure 11:**
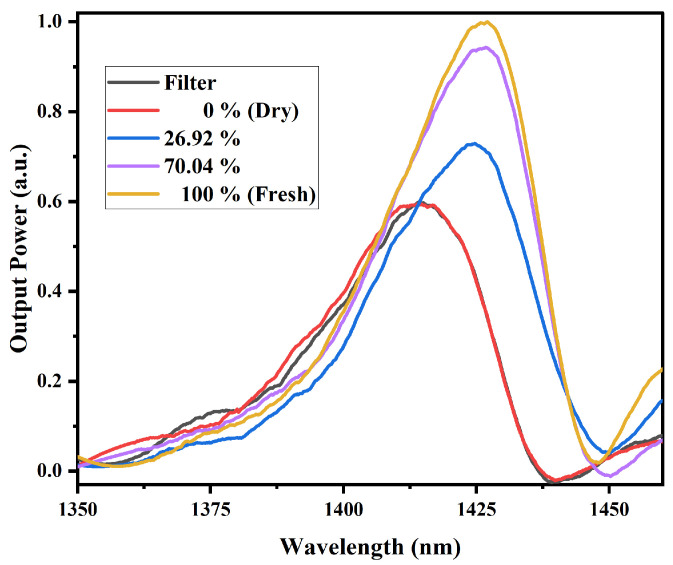
Spectral response of mango samples, obtained with the MMI sensor with the superluminescent laser source. The dry and fresh samples correspond to 0% and 100%, respectively.

Figure 11 shows the measurements performed on mango slices with different water content, where 100% refers to fresh fruit and 0% refers to dried fruit. The standard deviation ranged from 0.0192 to 0.0292, 0.0112–0.0170, 0.0121–0.0134, and 0.0273–0.0486 for MR values of 100%, 70.04%, 26.92%, and 0%, respectively. These small deviations indicate the stability and reliability of the sensor, even considering minor experimental uncertainties. These measurements were carried out using the MMI sensor designed for the superluminescent laser diode as laser source. The results show a clear increase in output power with increasing water content. Additionally, a shift of the spectrum to the right is also observed, which correlates with the moisture level in the fruit, indicating the sensor’s sensitivity to refractive index variations induced by water content.

**Figure 12:**
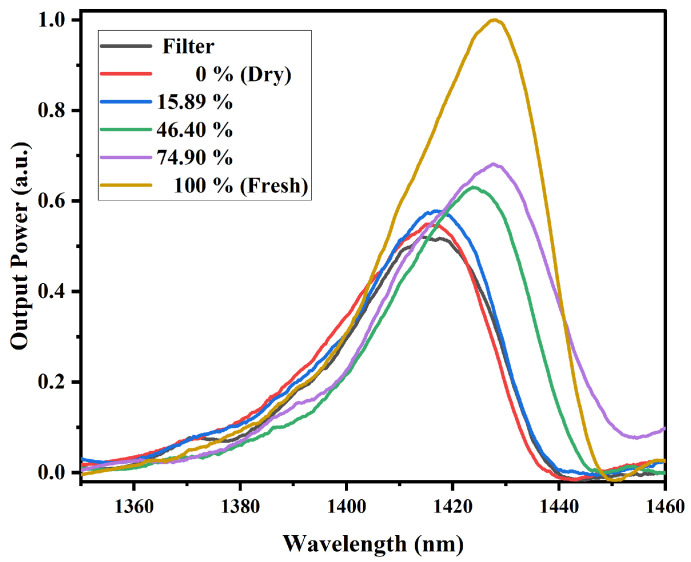
Spectral response of papaya samples, obtained with the MMI sensor with the superluminescent laser source. The dry and fresh samples correspond to 0% and 100%, respectively.

For the papaya sample (Figure 12), a behavior similar to that of the mango sample is observed. In this case, for MR values of 100%, 74.90%, 46.38%, 15.88%, and 0%, a standard deviation of 0.0191–0.0294, 0.0134–0.0209, 0.0111–0.0167, 0.0112–0.0169, and 0.018–0.0293, respectively, was obtained, respectively. The low standard deviation values confirm that the MMI-based sensor exhibits stable and repeatable output performance. As the water content in the fruit increases, the output power also increases, and the spectrum shifts to the right.

For this MMI sensor configuration, both fruit samples exhibit a similar behavior, where as the water content in the fruit increases, the output power also increases. Unlike the MMI sensor at 980 nm, in this case, a spectral shift is observed as the water content increases. The attenuation of the sensor output power could also depend on the intrinsic characteristics of the fruit slices, including their initial water and sugar content, as well as their structural composition. Water strongly absorbs in the NIR region due to vibrational overtones of the O–H bond, while sugars such as glucose and fructose exhibit weaker but noticeable absorption bands associated with C–H and O–H bonds. Moreover, the microstructure of the fruit, specifically the distribution of fibers and cellular integrity, likely affects light scattering within the sample, modifying the overall transmission detected by the MMI sensor [43,44].

It is important to remember that in NCF, light propagates within the cladding through MMI. Since there is no core, the evanescent field of these modes penetrates significantly into the surrounding medium. Therefore, the refractive index of the environment surrounding the NCF directly influences the modal distribution and the transmitted intensity detected at the output. When the external medium is air (n≈1.0003), the refractive index difference with the silica cladding (n≈1.444) is large. This results in strong optical confinement within the glass, a weak evanescent field, and consequently a greater modal mismatch at the SMF/NCF interfaces. Such mismatch increases scattering and coupling losses, which manifest in the transmission spectrum as lower transmitted power. Conversely, when the external medium is water (n≈1.33), the refractive index contrast decreases, allowing the evanescent field to extend further outside the fiber. This alters the multimode interference conditions and can enhance the phase matching among the reflected modes within the NCF. As a result, an increase in transmitted power can be observed, as seen in the experimental spectra [25,27,37,38]. During the dehydration process of the sample, the water content decreases while the fraction of dry components (cellulose, sugars, pectins) increases, leading to a higher local refractive index (n≈ 1.45–1.55). This causes an increase in the effective refractive index of the surrounding medium. Although the refractive index difference between the cladding and the environment tends to decrease, the detected power depends on both the evanescent coupling and the modal interference variations. In our results, the sensor in air exhibits lower transmitted power than with the dried fruit, indicating that despite the loss of moisture, evanescent coupling still occurs, improving modal matching compared to air, though to a lesser extent than in the fresh fruit [45,46,47]. Furthermore, the spectra obtained with both types of sensors show a remarkable difference: those recorded with the 980 nm laser source exhibit only variations in output power, while those obtained with the superluminescent laser source display, in addition to output power changes, a wavelength shift. As mentioned above, in both cases variations in the refractive index of the samples are detected (observed as changes in output power). However, this difference in spectral behavior indicates that, although in both sensors these refractive index variations are related to the water content in the fruit (changes in output power), the additional spectral shift observed with the superluminescent source also reflects sensitivity to structural changes in the water molecules within the samples due to the presence of sugars. This is consistent with the NIR absorbance spectrum, where strong absorption bands corresponding to water–molecule interactions are located around 970 nm, and bands associated with both water and sugar interactions appear around 1450 nm.

## 4. Conclusions

The results obtained in this study demonstrate the feasibility of using multimode interference (MMI)-based optical fiber sensors to monitor the water content in fruits such as mango and papaya during the drying process. A correlation was established between the water content of the samples and the optical output power of the sensor, attributed to variations in the refractive index of the samples as moisture is lost.

By employing two laser sources with wavelengths near 980 nm and in the 1400–1650 nm range, it was possible to distinguish between refractive index changes caused by water loss and those associated with structural alterations in water molecules due to the presence of sugars.

This work represents the first stage in the development of a portable, low-cost sensor capable of operating at temperatures up to 60 °C—an essential condition for in situ, real-time monitoring inside the drying chamber. However, before achieving this goal, it is necessary to establish the experimental and calibration conditions required to perform reliable quantitative measurements, since at its current stage, the sensor serves as a complementary tool to other techniques used for monitoring the fruit drying process.

Overall, these findings suggest that MMI-based sensors constitute a promising, non-destructive, and versatile tool for real-time monitoring of moisture and compositional changes in agricultural and food processing applications.

## Figures and Tables

**Table 1:** Parameters of the mathematical models.

	Mango			Papaya		
Model	Coef.	RMSE	R^2^	Coefficients	RMSE	R^2^
Lewis	k = 0.0137	0.0294	0.993	k = 0.0128	0.0459	0.984
Page	k = 0.00697	0.0219	0.996	k = 0.00324	0.0217	0.996
	n = 1.1562			n = 1.3166		
HP	k = 0.0140	0.0299	0.993	k = 0.0136	0.0437	0.986
	a = 1.0184			a = 1.0524		
Log	k = 0.0133	0.0285	0.993	k = 0.0126	0.0408	0.988
	a = 1.0336			a = 1.0774		
	c = −0.0206			c = −0.0331		

## Data Availability

Restrictions apply to the datasets. The datasets presented in this article are not readily available because they are part of an ongoing study. Requests to access the datasets should be directed to the corresponding author.

## Abbreviations

The following abbreviations are used in this manuscript:

SMF	Single Mode Fiber
MMF	Multimode Fiber
NCF	No-Core Fiber
MMI	Multimodal Interference
